# Signatures of cell stress and altered bioenergetics in skin fibroblasts from patients with multiple sclerosis

**DOI:** 10.18632/aging.103612

**Published:** 2020-07-08

**Authors:** Jordan M. Wilkins, Oleksandr Gakh, Parijat Kabiraj, Christina B. McCarthy, W. Oliver Tobin, Charles L. Howe, Claudia F. Lucchinetti

**Affiliations:** 1 Department of Neurology, Mayo Clinic, Rochester, MN 55905, USA; 2 Department of Immunology, Mayo Clinic, Rochester, MN 55905, USA; 3 Center for Multiple Sclerosis and Autoimmune Neurology, Mayo Clinic, Rochester, MN 55905, USA

**Keywords:** multiple sclerosis, skin fibroblasts, cellular stress, bioenergetics, metabolism

## Abstract

Multiple sclerosis (MS) is a central nervous system inflammatory demyelinating disease and the most common cause of non-traumatic disability in young adults. Despite progress in the treatment of the active relapsing disease, therapeutic options targeting irreversible progressive decline remain limited. Studies using skin fibroblasts derived from patients with neurodegenerative disorders demonstrate that cell stress pathways and bioenergetics are altered when compared to healthy individuals. However, findings in MS skin fibroblasts are limited. Here, we collected skin fibroblasts from 24 healthy control individuals, 30 patients with MS, and ten with amyotrophic lateral sclerosis (ALS) to investigate altered cell stress profiles. We observed endoplasmic reticulum swelling in MS skin fibroblasts, and increased gene expression of cell stress markers including *BIP*, *ATF4*, *CHOP*, *GRP94*, *P53*, and *P21*. When challenged against hydrogen peroxide, MS skin fibroblasts had reduced resiliency compared to ALS and controls. Mitochondrial and glycolytic functions were perturbed in MS skin fibroblasts while exhibiting a significant increase in lactate production over ALS and controls. Our results suggest that MS skin fibroblasts have an underlying stress phenotype, which may be disease specific. Interrogating MS skin fibroblasts may provide patient specific molecular insights and aid in prognosis, diagnosis, and therapeutic testing enhancing individualized medicine.

## INTRODUCTION

Multiple sclerosis (MS) is a central nervous system (CNS) inflammatory demyelinating disease characterized by focal inflammation, gliosis, and variable axonal injury [1]. Most patients present with relapses or neurological dysfunction related to focal inflammatory demyelinated lesions, however the majority eventually develop a progressive clinical course due to accumulating neurodegeneration. To date, genome-wide association studies have identified over 200 genetic variants that correlate with increased risk for MS highlighting the complexity of the disease [2]. Aging is often considered the greatest risk factor for neurodegenerative diseases resulting in genomic instability and epigenetic changes, cellular senescence, mitochondrial dysfunction, altered proteostasis, and deregulated nutrient sensing [3–5]. Indeed, several related biological processes have been reported to be altered in MS including cellular stress pathways, metabolism, senescence, and inflammation [6–10]. Additionally, environmental factors are also associated with increased risk for developing MS including diet, exercise, smoking, and vitamin D [11]. Therefore, MS is likely to be a multifactorial disease driven by genetics and the environment.

Studies investigating various biological fluids and tissues including cerebrospinal fluid, blood, urine, immune cells, brain, muscle, and the heart from patients with MS have reported abnormal function when compared to healthy controls [6, 11–16]. These findings may suggest that MS is a systemic disease. Therefore, utilization of primary cells from MS patients may provide insight into the pathophysiological mechanisms of the disease. Skin fibroblasts are a convenient source of primary cells as they are a peripheral tissue that is patient specific, can be cultured, differentiated into neural cell lines, and tested against therapeutics. Several lines of evidence suggest that skin fibroblasts derived from patients with neurodegenerative diseases have altered phenotypes when compared to healthy controls [17–20]. While studies using skin fibroblasts in neurodegenerative diseases including Alzheimer’s disease (AD), Parkinson’s disease (PD), Huntington’s disease (HD), and amyotrophic lateral sclerosis (ALS) have been investigated [17–20], perturbed function in MS skin fibroblasts remain largely unexplored.

In skin fibroblasts derived from patients with AD, mitochondrial abnormalities including altered morphology and production of reactive oxygen species (ROS) were reported [20]. Additional studies using AD skin fibroblasts detected reduced levels of protein kinase C, which was associated with altered levels of amyloid beta [21]. More so, early studies using AD skin fibroblasts reported increased production and deposition of amyloid beta [22, 23], which is a hallmark of AD in the brain. In PD, skin fibroblasts had altered growth properties in culture, increased ROS production, were more susceptible to UV radiation damage, and had altered mitochondrial function [18]. Skin fibroblasts derived from HD patients have reduced growth rates in culture, decreased ATP levels, and altered mitochondrial metabolic activity [19]. Patient skin fibroblasts from individuals with ALS also have altered cellular properties when compared to controls including hypermetabolic features, increased mitochondrial membrane potential and decreased mitochondrial content, reduced mitochondrial coupling, and proteome alterations [17, 24, 25]. These findings suggest that primary skin fibroblasts from patients with neurodegenerative disorders retain inherent abnormalities that perturb cellular homeostasis and bioenergetics.

Based on observations in biospecimens from patients with MS and studies in skin fibroblasts from individuals with neurodegenerative diseases [6, 11–15, 17–20], we hypothesized that inherent changes associated with MS may alter cell stress pathways and the bioenergetics in patient-derived skin fibroblasts. Our findings indicate that MS skin fibroblasts have an underlying stress phenotype, which likely results in a predisposition to metabolic dysfunction and altered bioenergetics. Furthermore, comparison of changes in MS skin fibroblasts to those derived from patients with ALS would suggest that these alterations are to some extent disease specific. Findings from our study suggest that MS skin fibroblasts may be suitable for the development of biomarkers and aid in the prognosis, diagnosis, and testing of therapeutic treatments. Additionally, as skin fibroblasts are patient-specific, advancements in the characterization of altered stress phenotypes and bioenergetics may help advance individualized medicine.

## RESULTS

### Human skin fibroblasts used in study

In this study, skin fibroblasts were harvested from patients diagnosed with MS or ALS, and control individuals with no apparent neurological disorders (Supplementary Table 1). At the time of skin fibroblast harvest, 27 patients were diagnosed with relapsing-remitting MS (RRMS), two with secondary progressive MS (SPMS), and one with clinically isolated syndrome (CIS). The median age of skin fibroblasts at harvest for controls was 46 years (SD = 15.1, % female = 46), MS was 47.5 years (SD = 13.2, % female = 63), and ALS was 53 years (SD = 13.9, % female = 50). It is worth noting that diagnosis of MS usually occurs between the ages of 20 and 50, while ALS ranges from 40 to 65, contributing to the increased median age in ALS skin fibroblasts used in this study. For MS individuals, disease duration from time of diagnosis to skin fibroblast harvest ranged from 0.1 to 29.3 years, while in ALS the duration ranged from 0.9 to 6.1 years (Supplementary Table 1).

### ER stress is increased in MS skin fibroblasts

**Figure 1:**
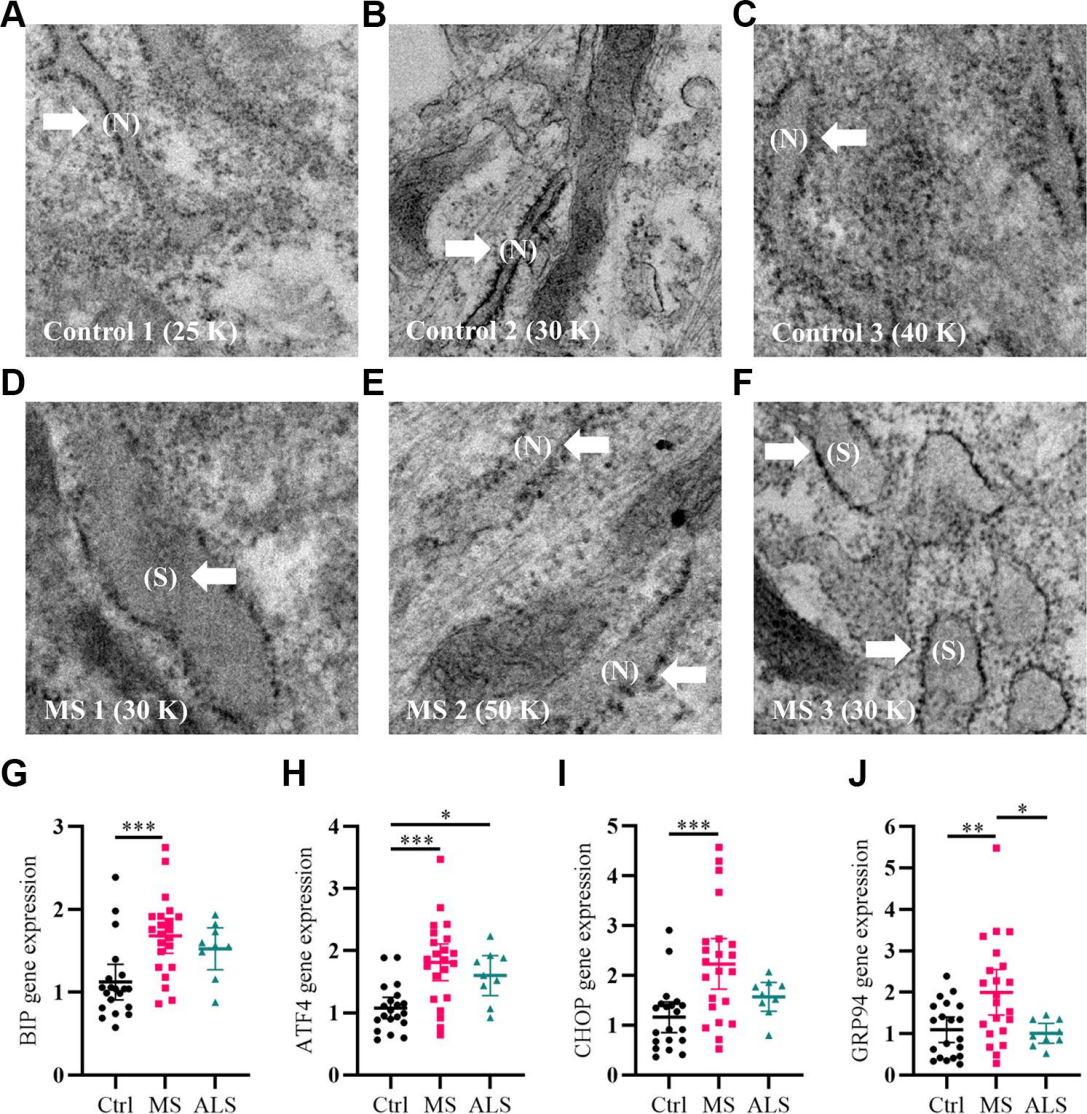
**Signatures of ER stress are increased in MS skin fibroblasts.** (**A**–**F**) Skin fibroblasts were imaged using electron microscopy. Representative images of three different skin fibroblasts from (**A**–**C**) control and (**D**–**F**) MS individuals are shown. Arrows point to (N, normal) or (S, swollen) ER. (**G**–**J**) Gene expression levels of *BIP*, *ATF4*, *CHOP*, and *GRP94* were determined using RT-PCR. Detected gene expression levels were normalized to the reference gene β-actin. Each point represents a unique skin fibroblast (Ctrl, n = 20; MS, n = 22; ALS, n = 9). The average of replicates is shown with the 95% confidence interval. Significance between groups was determined using one-way ANOVA post hoc Tukey test. *, P < 0.05; **, P < 0.01; and ***, P < 0.001. Abbreviations: ALS, amyotrophic lateral sclerosis; Ctrl, control; ER, endoplasmic reticulum; MS, multiple sclerosis; N, normal; S, swollen.

As a first step, we qualitatively analyzed the cellular structure and organization of MS skin fibroblasts using electron microscopy (EM) compared to healthy controls. An increase in endoplasmic reticulum (ER) swelling was more apparent in MS skin fibroblasts when compared to controls (Figure 1A–1F). Whereas the ER in control cell lines remained predominately long and narrow (Figure 1A–1C), we observed a substantial increase in the rounding and swelling of the ER (Figure 1D–1F) in two of the three MS skin fibroblasts. Swelling of the ER is commonly associated with increased cellular stress and the activation of the unfolded protein response (UPR) pathway [26]. Thus, we used quantitative PCR to measure the gene expression level of multiple UPR markers in skin fibroblasts from MS, ALS, and control individuals (Figure 1G–1J). Compared to control skin fibroblasts, MS cells had significant increases in the gene expression level of *BIP* (heat shock protein family A member 5, HSPA5/BIP, P = 0.0006), *ATF4* (activating transcription factor 4, P = 0.0001), *CHOP* (DNA damage inducible transcript 3, DDIT3/CHOP, P = 0.0008), and *GRP94* (heat shock protein 90 beta family member 1, HSP90B1/GRP94, P = 0.0077) (Figure 1G–1J). Likewise, the gene expression level of *GRP94* was significantly higher in MS when compared to ALS fibroblasts (P = 0.0256, Figure 1J). In ALS skin fibroblasts, *ATF4* gene expression level was significantly higher when compared to controls (P = 0.0461, Figure 1H). These results suggest that MS skin fibroblasts have an underlying stress phenotype that may be distinct from normal controls and ALS.

### Altered gene expression of cell stress markers in MS skin fibroblasts

**Figure 2:**
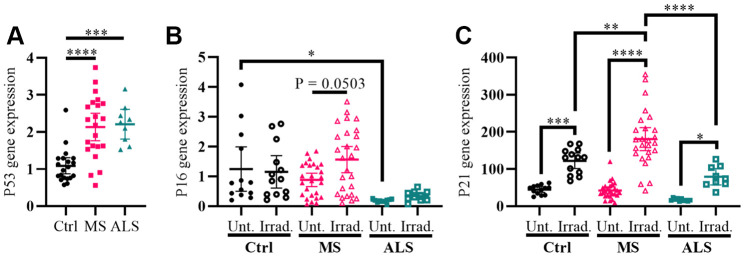
**Markers *P53* and *P21* have altered profiles in MS skin fibroblasts.** (**A**) Skin fibroblasts were cultured in standard media conditions for one week. Total RNA was harvested and the gene expression level of *P53* was determined by RT-PCR (Ctrl, n = 20; MS, n = 22; ALS, n = 9). The relative fold change compared to the average of all controls is shown. (**B** and **C**) Skin fibroblasts were seeded and cultured for 24 hours in standard media conditions (Ctrl, n = 13; MS, n = 26; ALS, n = 10). The cells were either untreated or treated with gamma irradiation and incubated for an additional ten days prior to harvesting total RNA. The gene expression levels of *P16* and *P21* were determined using RT-PCR. (**A**–**C**) All gene expression levels were normalized to the TATA box protein gene. Each data point represents a unique cell line. The average of replicates is shown with the 95% confidence interval. Significance was determined using one-way ANOVA post hoc Tukey test. *, P < 0.05; **, P < 0.01; ***, P < 0.001; ****, P < 0.0001. Abbreviations: ALS, amyotrophic lateral sclerosis; Ctrl, control; Irrad, irradiated; MS, multiple sclerosis; Unt, untreated.

Cellular stress including oxidative stress, DNA damage, epigenetic modifications, oncogene activation, ionizing radiation, and hypoxia can modulate the activity of cell cycle regulators in order to help maintain homeostasis [27–29]. The genes *P53* (tumor protein P53), *P16* (cyclin dependent kinase inhibitor 2A, CDKN2A/P16), and *P21* (cyclin dependent kinase inhibitor 1A, CDKN1A/P21) are key cell cycle regulators and are increasingly recognized for their role in aging, senescence, oxidative stress, inflammation, and neurodegenerative diseases including MS [27–33]. To further evaluate the presence of increased cellular stress in MS skin fibroblasts, we measured the gene expression levels of *P53*, *P16*, and *P21* using quantitative PCR, which was compared to ALS and control individuals (Figure 2). We detected a significant increase in the *P53* gene expression level in both MS and ALS skin fibroblasts (P < 0.0001 and P = 0.0003, respectively) compared to controls (Figure 2A). Compared to control fibroblasts, the endogenous level of *P16* in ALS individuals was significantly lower (P = 0.0392, Figure 2B). When exposed to gamma irradiation, a common inducer of senescence and cell stress [34, 35], we detected a near significant increase of *P16* in MS skin fibroblasts (P = 0.0503, Figure 2B) while minimal changes were observed in ALS and controls. The endogenous *P21* gene expression level between MS, ALS, and control skin fibroblasts was similar (Figure 2C). After gamma irradiation treatment, we detected significant increases of *P21* gene expression in all skin fibroblasts when compared to their respective untreated groups (P_ctrl_ = 0.0004, P_MS_ < 0.0001, P_ALS_ = 0.0384, Figure 2C). Furthermore, the *P21* gene expression level in irradiated MS skin fibroblasts was significantly higher compared to the treated ALS (P < 0.0001) and control (P = 0.003) groups (Figure 2C). These results further reflect an inherent cellular stress phenotype in MS skin fibroblasts, which may be disease specific.

### MS skin fibroblasts have reduced resiliency to oxidative stress

**Figure 3:**
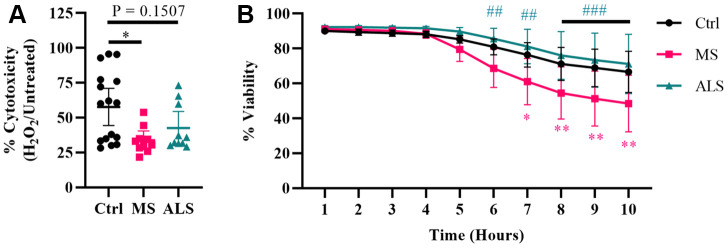
**Skin fibroblasts from MS patients have reduced resiliency.** Skin fibroblasts were cultured in standard media for 24 hours prior to the assay. (**A**) Cells were treated with 200 μM hydrogen peroxide for two hours followed by incubation with MTT reagent for an additional two hours (Ctrl, n = 16; MS, n = 10; ALS, n = 10). All cells were lysed and the resulting formazan crystals were solubilized prior to measurement. The percent cytotoxicity was determined using a ratio between treated and untreated cells. Each data point represents a unique cell line and is the average of all measurements. The 95% confidence interval is shown. Significance was determined using one-way ANOVA post hoc Tukey test. (**B**) Skin fibroblasts were treated with 200 μM hydrogen peroxide for ten hours (Ctrl, n = 13; MS, n = 8; ALS, n = 10). Fluorescence data using the CellTox Green assay was measured every hour. Eight unique cell lines per group were used. Percent viability is relative to the one hour time reference. The data represents the average of all measurements with the 95% confidence interval shown. Significance was determined using two-way ANOVA post hoc Tukey test. In (**B**), * indicates significance between Ctrl and MS, and # for MS and ALS. No significance was determined between Ctrl and ALS. *, P < 0.05; **, P < 0.01; ***, P < 0.001. Abbreviations: ALS, amyotrophic lateral sclerosis; Ctrl, control; H_2_O_2_, hydrogen peroxide; MS, multiple sclerosis.

Evidence suggests that mitochondrial dysfunction is a key driver in the pathology of MS [36]. Mitochondrial damage is thought to increase the production of ROS resulting in oxidative stress and injury to the surrounding tissue in MS [36, 37]. Additionally, oxidative damage can result in ER and cellular stress [30, 31, 33, 38]. Given that MS skin fibroblasts show underlying signatures of cellular stress, we sought to determine if they have an altered ability to manage exogenous stress. To do so, we treated MS, ALS, and control skin fibroblasts with hydrogen peroxide and monitored their resiliency using two different cell stress assays (Figure 3). We first tested the cytotoxic response of the skin fibroblasts against an acute treatment with hydrogen peroxide using the MTT assay (Figure 3A). Cells were plated in standard media conditions followed by a two hour treatment with 200 μM hydrogen peroxide. We found that MS skin fibroblasts had a significantly reduced resiliency to hydrogen peroxide treatment when compared to controls (P = 0.0129, Figure 3A). Since the MTT assay reflects metabolic activity of a cell and may not necessarily represent viability [39], we further tested the skin fibroblasts against hydrogen peroxide using the CellTox Green assay. The CellTox Green assay utilizes a membrane-impermeable fluorescent molecule, which produces an increased signal upon binding to DNA. Therefore, the CellTox Green assay more closely detects non-viable cells with compromised membranes [40]. To measure cell viability, skin fibroblasts were treated with hydrogen peroxide and monitored hourly out to ten hours (Figure 3B). Compared to control cells, MS skin fibroblasts displayed greater cytotoxicity by seven hours post-treatment with hydrogen peroxide (P = 0.0132, Figure 3B), which remained significantly different out to ten hours (P = 0.0024, Figure 3B). Furthermore, ALS fibroblasts behaved similarly to controls and did not significantly differ out to ten hours of treatment (Figure 3B). Taken together, these results suggest that both MS and ALS skin fibroblasts have a reduced resiliency to manage exogenous stress compared to control cells (Figure 3A). However, given MS skin fibroblasts have reduced viability compared to ALS and control cells when treated with hydrogen peroxide (Figure 3B), our findings may suggest cell survival mechanisms are altered in MS fibroblasts and may be disease specific.

### Metabolic function in MS skin fibroblasts is perturbed

**Figure 4:**
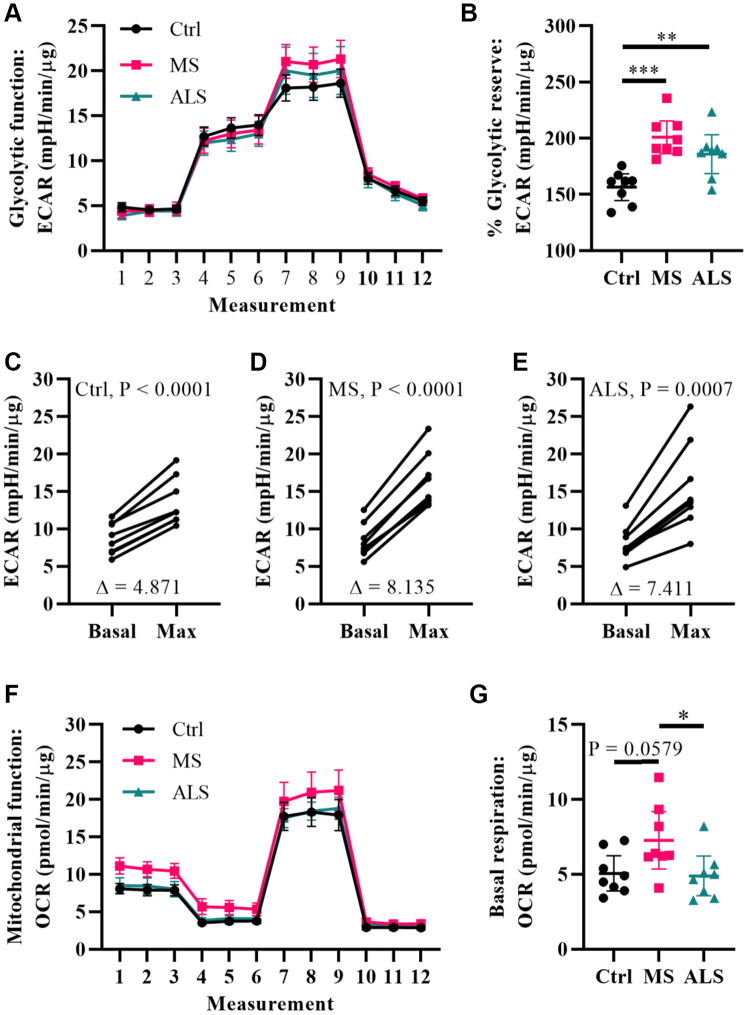
**Multiple sclerosis skin fibroblasts have altered bioenergetics.** (**A**) Glycolytic function of MS, ALS, and control skin fibroblasts were assayed using the Seahorse Glycolytic Stress Test. The cells were incubated in glucose-free media containing 2 mM glutamine for one hour prior to measuring glycolytic function. Sequential addition of 5.5 mM glucose, 1.5 μM oligomycin, and 50 mM 2-deoxy-D-glucose were added to the cells while the ECAR was monitored. (**B**) The percent glycolytic reserve (glycolytic capacity/basal glycolysis) increase after treatment with oligomycin. (**C**–**E**) Paired analysis of skin fibroblasts treated with oligomycin reflects individual changes from basal glycolysis to maximum glycolytic capacity. The average change (**Δ**) is indicated. (**F**) Mitochondrial function of MS, ALS, and control skin fibroblasts were assayed using the Seahorse Mito Stress Test. All cells were incubated in assay medium containing 5.5 mM glucose and 2 mM glutamine for one hour prior to performing the assay. The OCR was monitored during sequential injection of 1.5 μM oligomycin, 1 μM FCCP, and 1 μM rotenone plus 1μM antimycin A. (**G**) The average basal respiration detected in skin fibroblasts. (**A** and **F**) Each dot represents the average rates measured ± SEM from all eight unique cell lines within each indicated group. (**B** and **G**) Each dot represents a unique patient-derived skin fibroblast (n = 8 per group). The graphs show the average with the 95% confidence interval. Statistical significance was determined using one-way ANOVA post hoc Tukey test. *P < 0.05, **P < 0.01, ***P < 0.001. (**C**–**E**) Significance was determined using paired t test analysis (two tailed) using averaged data. All averages were determined from two independent experiments each containing triplicates using all three measurements per injection group. All data was normalized to total protein content (μg).

Metabolic processes are highly dynamic and are known to change in response to cellular demands and stress [41]. Several reports have associated mitochondrial dysfunction and metabolic alterations in MS [36, 42, 43]. Thus, we predicted that the stress phenotype detected in MS skin fibroblasts may result in a predisposition to metabolic dysfunction. To gain insight whether the bioenergetics in MS skin fibroblasts were altered, we utilized Seahorse technology to monitor cellular respiration and extracellular acidification (Figure 4). The Seahorse analyzer monitors oxygen consumption rate (OCR) and extracellular acidification rate (ECAR) to provide insight into the metabolic function of live cell cultures [44]. The OCR reflects activity of oxidative phosphorylation where oxygen is consumed during the generation of ATP [44]. Similarly, ECAR is used to monitor glycolytic activity by measuring pH changes presumably due to the production of lactate, an endpoint product of glycolysis [44]. The bioenergetics profiles of skin fibroblasts from MS and ALS individuals were compared to controls and monitored for differences in ECAR (Figure 4A–4E) and OCR (Figure 4F and 4G) using standard Seahorse conditions. Differences in the glycolytic function of MS cells were detected when compared to controls (Figure 4A). In particular, when the skin fibroblasts were treated with oligomycin (an inhibitor of ATP synthase), we detected the greatest percent glycolytic reserve increase in MS skin fibroblasts when compared to ALS and control cells (Figure 4B). As expected, all skin fibroblasts increased their glycolytic flux upon treatment with oligomycin (Figure 4C–4E). However, the average oligomycin-induced increase in glycolytic flux was detected in MS cells (Δ8.14, Figure 4D) compared to ALS (Δ7.41, Figure 4E) and control (Δ4.87, Figure 4C) skin fibroblasts. This suggests that both MS and ALS skin fibroblasts undergo greater metabolic alterations when mitochondrial function is perturbed, which may rely on increased glycolytic functions when compared to control cells. Using the Seahorse Mitochondrial Stress Test, we further analyzed mitochondrial function in MS, ALS, and control skin fibroblasts (Figure 4F). In MS skin fibroblasts, we detected a near significant increase in basal oxygen consumption (P = 0.0579) when compared to controls (Figure 4G). A significant increase in MS basal oxygen consumption (P = 0.0393) was detected when compared to ALS skin fibroblasts (Figure 4G). These results are indicative of altered mitochondrial function in MS skin fibroblasts when compared to ALS and control cells. Taken together, these results demonstrate that the bioenergetics in MS and ALS skin fibroblasts are altered compared to control cells but may be disease specific.

### Metabolomics profiling of MS skin fibroblasts

To gain further insight into alterations in the bioenergetics of MS skin fibroblasts, we utilized quantitative targeted metabolomics to measure metabolites of glycolysis, the TCA cycle, and intermediates associated with mitochondrial metabolism (Table 1). Skin fibroblasts from MS and controls were cultured in standard media for 24 hours. The spent media was analyzed by GC-MS and total protein content was used to normalize metabolite concentrations. A total of 17 metabolites were measured (Table 1). A two-tailed t test indicated five metabolites to be significantly increased (P < 0.05 and FDR < 0.2) in the media from MS skin fibroblasts compared to the controls (Table 1). Two metabolites are associated with the TCA cycle including malic and 2-ketoglutaric. Interestingly, the remaining three metabolites (2-ketoisovaleric, 2-ketoisocaproic, and 3-methyl-2-ketovaleric) are forms of keto acids associated with maple syrup urine disease (MSUD), a metabolic disorder [45]. Two additional metabolites trended towards being significantly increased in MS media including lactate (P = 0.062), an end product of glycolysis, and succinic (P = 0.072) a TCA cycle intermediate (Table 1).

**Table 1:** Targeted metabolomics in MS skin fibroblasts.

**Characteristic**	**Control (n = 8)**		**Multiple sclerosis (n = 8)**		**Two-sample t-test/Fishers exact test**	**FDR**
Age	50.1	(14.9)	42.6	(7.6)	0.22	
Sex (male/female)	3/5		4/4		1.0	
Malic	107	(24)	179	(51)	0.005	0.08
2-Ketoisovaleric	120	(57)	204	(54)	0.009	0.08
2-Ketoisocaproic	349	(82)	531	(163)	0.018	0.1
2-Ketoglutaric	53	(10)	74	(21)	0.028	0.12
3-Methyl-2-ketovaleric	120	(53)	182	(54)	0.036	0.12
Lactic	89946	(20544)	116123	(29755)	0.062	0.18
Succinic	1371	(309)	1885	(655)	0.072	0.18
cis-Aconitic	20	(4)	35	(22)	0.101	0.21
Fumaric	51	(11)	66	(22)	0.115	0.22
Citric	637	(138)	806	(278)	0.153	0.26
3-OH Isovaleric	108	(39)	91	(30)	0.318	0.49
Acetoacetic	31	(14)	42	(35)	0.419	0.54
Pyruvic	1727	(453)	1937	(582)	0.434	0.54
3-OH Butyric	539	(116)	651	(373)	0.441	0.54
2-Ketobutyric	164	(41)	183	(62)	0.487	0.55
3-Methylglutaconic	21	(7)	23	(7)	0.543	0.58
2-OH Butyric	82	(26)	90	(54)	0.736	0.74

Targeted metabolomics analysis of conditioned media from skin fibroblasts derived from control and multiple sclerosis individuals. The mean value for each characteristic is shown where applicable with the standard deviation in parentheses. The average metabolite concentration was determined from eight different patient skin fibroblasts per group tested in quadruplicates. Metabolite concentrations are shown as μmol of metabolite per g of protein. Significance for group comparisons was calculated using two-sample t-test (two-tailed) and Fisher's exact test for categorical variables. Metabolites are ordered by p-value. FDR: false discovery rate.

### Lactate production is increased in MS skin fibroblasts

**Figure 5:**
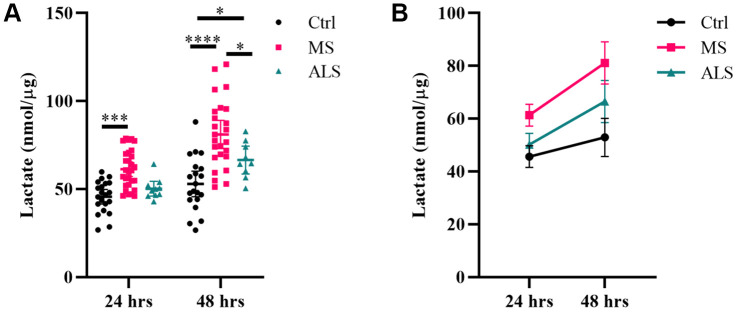
**Lactate production is increased in MS skin fibroblasts.** Skin fibroblasts from MS (n = 25-29), ALS (n = 9-10), and control cells (n = 20-21) were incubated in media (without FBS) for 24 hours or 48 hours prior to measuring lactate concentrations in the media. (**A**) Significant changes between groups were determined using two-way ANOVA post hoc Tukey test (repeated measures with mixed model). Each data point represents a unique skin fibroblast sample. Determined lactate concentrations were normalized to cellular protein content. The average of replicates is shown with the 95% confidence interval. (**B**) Summary data of detected lactate concentrations after 24 or 48 hours. The average of all data is shown per group with the 95% confidence interval. *, P < 0.05, ***, P < 0.001; ****, P < 0.0001. Abbreviations: ALS, amyotrophic lateral sclerosis; Ctrl, control; MS, multiple sclerosis.

Given we saw increased glycolytic flux in MS skin fibroblasts treated with oligomycin (Figure 3B), and a near significant increase in lactate in MS conditioned media (Table 1), we sought to further evaluate if lactate production is increased in MS skin fibroblasts compared to controls. Utilizing a Lactate Assay Kit, we measured the lactate concentration in the spent media from cells cultured in two different compositions (with and without FBS). In the presence of FBS, we detected increased lactate production in MS cells compared to ALS and control cells (Supplementary Figure 1). However, since FBS can contain lactate and enzymes including lactate dehydrogenase, we performed an extended assay in the absence of FBS (Figure 5). Skin fibroblasts from MS, ALS, and controls were cultured in the absence of FBS and the lactate concentration in the spent media was assayed after 24 and 48 hours (Figure 5). We found that MS skin fibroblasts generated a greater concentration of lactate in the media after 24 and 48 hours when compared to controls (P = 0.0003 and P < 0.0001, respectively, Figure 5A). Additionally, after 48 hours, MS skin fibroblasts had significantly more lactate in the media when compared to ALS cells (P = 0.0191, Figure 5A). After 48 hours, ALS skin fibroblasts also generated a greater amount of lactate compared to controls (P = 0.0301, Figure 5A). Overall, we detected greater rates of lactate production in MS skin fibroblasts compared to ALS and control cells (Figure 5B). This data suggests that flux through pathways that generate lactate, including glycolysis and glutaminolysis, may be increased in MS skin fibroblasts compared to both ALS and controls.

## DISCUSSION

The cause of MS is unknown and the development of biomarkers for the prognosis, diagnosis, and monitoring of therapeutic efficacy remains a challenge. While animal models of MS (e.g. experimental autoimmune encephalomyelitis (EAE), Theiler's murine encephalomyelitis virus, toxin-induced demyelination, cuprizone model, etc.) have been instrumental in the understanding of pathological mechanisms, they are limited by their ability to fully recapitulate the disease. Therefore, the development of additional models to study MS is necessary. Skin fibroblasts present a promising opportunity to study pathophysiological mechanisms of MS since they are patient-specific, retain genetic variations associated with the disease, which may include epigenetic modifications, and are a readily available peripheral tissue that can be cultured. Furthermore, skin fibroblasts can be utilized for induced pluripotent stem cell (iPSC) technology allowing for the differentiation of cells into oligodendrocytes, neurons, and astrocytes. In MS, aging, increased cellular stress, dysregulation of mitochondria, and altered bioenergetics are associated with the pathogenesis and progression of the disease [3–10]. Given that the etiology of MS is likely heterogeneous and triggered by several factors [46], continued identification and characterization of altered cellular functions in models of MS will be necessary to establish reliable biomarkers and understand disease mechanisms. Towards this end, we utilized skin fibroblasts derived from patients with MS, ALS, and healthy controls to determine if we could detect signatures of cell stress and altered bioenergetics and whether these were disease specific.

Several forms of cellular stress are likely to contribute towards the progression of MS including inflammation, oxidative stress, mitochondrial dysfunction, and altered bioenergetics. Disruption of normal cellular homeostasis can result in the activation of several cell stress response pathways. In MS skin fibroblasts, we observed an increase in ER swelling indicative of cell stress. Activation of the UPR pathway is one of the most well-known mechanisms during increased ER stress [47]. Indeed, we found elevated gene expression markers of the UPR pathway in MS skin fibroblasts including *BIP*, *ATF4*, *CHOP*, and *GRP94*. The chaperones BIP and GRP94 function in the regulation of protein folding, cell survival, calcium storage, and immune function [48]. Sustained ER stress can trigger cell death through ATF4 and CHOP signaling [49]. The UPR is a complex signal transduction pathway regulated by at least three key factors including PERK (protein kinase RNA-like ER kinase), IRE1 (inositol-requiring protein 1), and ATF6 (activating transcription factor 6) [50]. In non-stressed conditions, chaperones are bound to these three factors preventing their activity. During ER stress, the chaperones dissociate from PERK, IRE1, and ATF6 enhancing their activity in an attempt to establish ER and cellular homeostasis [50]. In the CNS, proper maintenance of the ER is crucial for myelin biosynthesis [51]. In MS active lesions, increased expression of UPR markers were detected in oligodendrocytes, astrocytes, microglia, and T cells [7]. In the EAE mouse model of MS, activation of the UPR-associated protein PERK by interferon gamma prevented demyelination, axonal damage, and loss of oligodendrocytes [52]. These studies highlight the potential importance of ER stress signaling in the pathogenesis of MS. Furthermore, the involvement of ER stress in multiple neurodegenerative diseases is well documented [53].

The genes *P53*, *P16*, and *P21* are crucial regulators of the cell cycle. Increased cellular stress including ER stress, mitochondrial dysfunction, oxidative stress, DNA damage, and inflammation can cause cell cycle arrest inducing senescence and/or apoptosis in order to prevent damaged cells from proliferating [28, 30–33]. Cellular arrest is closely associated with neurodegenerative diseases including MS [32, 33]. In demyelinated brain tissue of MS patients, an increased expression of P16 was detected in progenitor cells [32]. Additionally, neural progenitor cells derived from MS patient iPSCs had increased expression of P16 and P53 markers compared to controls [32]. Further evidence in a mouse model of MS suggests that the extent of demyelination is correlated with senescent cell load [54]. Taken together, these findings may suggest that modulation of cell cycle regulators can impact the remyelination process. In support of these findings, we detected a significant increase in the gene expression level of *P53* in MS skin fibroblasts compared to controls. While endogenous expression of *P16* and *P21* were similar in MS and control skin fibroblasts, treatment of the cells with gamma irradiation resulted in a greater increase of *P21* in MS cells compared to controls. Given the role of P53 in multiple cell stress responses [28], elevated *P53* gene expression in MS skin fibroblasts compared to controls may further indicate an increased stress phenotype. More so, P53 is known to activate P21 in situations of increased cellular stress, which can cause cell cycle arrest and senescence [27]. Senescent cells are known to alter their secretome referred to as the senescence-associated secretory phenotype (SASP), which increases the release of proinflammatory factors and may contribute towards the pathogenesis of MS [10]. Though the exact mechanisms remain to be elucidated, our results in skin fibroblasts further support a role for the cell cycle regulators P53, P16, and P21 in the pathogenesis of MS.

Normally, cells have multiple mechanisms to help restore homeostasis (e.g. antioxidant response pathways, UPR, senescence, etc.). When the extent of damage is beyond repair, additional pathways are engaged that trigger cell death. As we found signatures of heightened cellular stress in MS skin fibroblasts, we explored their ability to cope with exogenous cellular stress. Oxidative stress is highly implicated in MS [36, 37, 55]. Furthermore, ER stress and senescence can be triggered by oxidative stress [30, 31, 33, 38]. Therefore, we investigated the cytotoxic effects of hydrogen peroxide on MS skin fibroblasts. Compared to controls, we found that MS skin fibroblasts had reduced resiliency when challenged with hydrogen peroxide. Both the MTT assay and CellTox Green assay suggest that metabolic function and cell viability, respectively, are reduced in MS skin fibroblasts compared to controls when treated with hydrogen peroxide. This supports the notion that an underlying stress phenotype in MS skin fibroblasts renders them more susceptible to additional cytotoxic events.

Metabolism plays a key role in maintaining cellular homeostasis. Crosstalk between metabolism and cell stress pathways are continually being described. For instance, the ER is not only important for its role in stress responses, but also in the metabolic function of the cell (e.g. lipid metabolism). Similarly, mitochondria are involved in several processes including cell death, metabolic control, and energy production. Hence, increased cell stress can cause changes in metabolic function. Indeed, both ER stress and cellular arrest are linked to increased inflammation and altered metabolic function [56, 57]. Inflammation is a key driver in MS, and the involvement of mitochondria mediating inflammatory signaling and metabolic switching in immune cells is well recognized [58–60]. Therefore, we further investigated whether the stress phenotype detected in MS skin fibroblasts was associated with altered metabolic function. Using Seahorse technology, we monitored the glycolytic and mitochondrial metabolic function in the fibroblasts. We found that when cells were treated with oligomycin, there was a substantially larger increase in the percent glycolytic reserve of MS skin fibroblasts compared to controls. This may indicate that MS cells require greater glycolytic flux to compensate for decreased mitochondrial ATP synthase activity compared to control cells. Furthermore, MS skin fibroblasts had a near significant increase in basal mitochondrial respiration, which may indicate greater energetic demand commonly associated with increased cell stress. Interestingly, a recent study using T cells from patients with RRMS during a relapsing phase showed increased mitochondrial and glycolytic function when compared to remitting MS and healthy controls [61]. Taken together, our data suggests that the bioenergetics in MS skin fibroblasts is altered compared to controls.

To gain further insight into the altered bioenergetics of MS skin fibroblasts, we performed quantitative targeted metabolomics including metabolites of glycolysis, the TCA cycle, and keto acids. Interestingly, we found increases in multiple alpha-keto acids, which result from the catabolism of branched chain amino acids [45, 62]. Increased keto acid production is linked to the disorder MSUD, a neurometabolic disorder [45, 63]. The disease is characterized by a deficiency in the branched-chain alpha-keto acid dehydrogenase complex, which may contribute to increased oxidative stress in the brain [45, 63]. Treatment of glial cells with keto acids resulted in morphological changes, oxidative stress, and cellular death [64]. In the brain, increased keto acid production may be associated with alterations in oxidative metabolism resulting in energy failure and lactic acidosis [65, 66]. In addition to keto acids, we found increases in malate, alpha-ketoglutarate, and a near significant increase in lactate in the media from MS skin fibroblasts when compared to controls. These results may further suggest that disturbances exist in the lactate-malate-aspartate shuttle, which is important for maintaining NAD+/NADH ratios in the cell [66, 67]. Indeed, several studies have linked deficiencies in keto acid metabolism, lactate production, the malate-aspartate shuttle, and neurological deficits [66].

Lactate metabolism is now implicated in several biological processes including tumor formation [68], stress resistance and cell survival [69], mitochondrial biogenesis [70], epigenetics [71], and immune function [72]. Interestingly, increased lactate concentration was detected in the CSF and serum of individuals with MS compared to healthy controls [6, 16], which may be indicative of altered bioenergetics and mitochondrial dysfunction. In MS skin fibroblasts, we detected increased glycolytic function (percent glycolytic reserve) and a near significant increase in lactate production using metabolomics. Therefore, we further sought to determine if lactate production in MS skin fibroblasts was increased compared to controls. Indeed, using a Lactate Assay Kit, we detected a significant increase in lactate production from MS skin fibroblasts when compared to controls. Thus, it is plausible that increased lactate production in MS skin fibroblasts reflects underlying cellular stress, as it was detected in the CSF and serum from MS individuals [6, 16]. Furthermore, these results suggest that lactate may be able to distinguish between MS skin fibroblasts and healthy controls.

In this study, we sought to determine if changes detected in skin fibroblast derived from MS patients may be distinct from other neurodegenerative diseases. We included skin fibroblasts from patients with ALS, a progressive neurodegenerative disease affecting motor neurons. While we saw similar trends for some of the parameters measured in this study, there were distinct changes in MS skin fibroblasts compared to ALS and controls. These alterations included the markers GRP94 and P21, cell survival rates under oxidative stress, and likely metabolic function. Skin fibroblasts from ALS patients have previously been studied for altered function compared to control cells. In a study by Yang et al., inhibition of the ubiquitin-proteasome system and induction of oxidative stress in ALS skin fibroblasts resulted in ALS-linked cellular pathologies [73]. The authors further noted that these cellular stresses did not lead to increased cytotoxicity when compared to controls [73]. These observations are in line with our findings that cell survival was decreased in MS skin fibroblasts while remaining similar in ALS and control cells. Konrad and colleagues further reported a correlation between increased ECAR and lactate production in ALS skin fibroblasts when compared to control cells, though the extent of lactate increase was not shown [17]. In MS skin fibroblasts, lactate production was significantly increased compared to ALS and control cells after 48 hours in culture. At this point, ALS skin fibroblasts also had a significant increase compared to controls. Thus, it may be that MS skin fibroblasts have a greater dependency on glycolytic-related mechanisms to maintain homeostasis compared to ALS and control cells. Nonetheless, these results support the notion that skin fibroblasts from patients with neurodegenerative diseases can modulate their metabolism to maintain cellular homeostasis.

Taken together, we determined that the skin fibroblasts derived from MS patients have an underlying cell stress phenotype, which likely predisposes the cells to altered bioenergetics. Furthermore, comparison of MS skin fibroblasts to ALS cells demonstrates that these changes are likely disease specific. While any one single marker alone may not be useful, a combination of markers in the skin alongside other clinical parameters may help with earlier detection and treatment planning of MS. One limitation of studies using skin fibroblasts is the inability to obtain complete demographical data; future studies would benefit using additional correlative data such as disease status, medications used, whether or not the individuals smoke, etc. for both diseased and what we deem as healthy individuals. Additionally, genomics profiling would greatly enhance our understanding of the detected changes as patient specific genetic alterations are likely to contribute to variability within any given study. Nevertheless, our study demonstrates that skin fibroblasts could serve as an additional model to study MS branching important aspects that are missing from animal models. In conclusion, our findings suggest that patient derived cells may advance individualized medicine and aid in biomarker development for the prognosis and diagnosis of neurodegenerative diseases.

## MATERIALS AND METHODS

### Reagents and materials

Minimum essential medium (MEM; cat. # 10-010-CV), MEM without phenol red (cat. # 17-305-CV), phosphate-buffered saline (PBS, cat. # 21-031-CV), glucose solution (cat. # 25-037-CI), and MEM nonessential amino acids (NEAA, cat. # 25-025-CI) were purchased from Corning, (New York, NY, USA). Dulbecco’s modified eagle medium without phenol red (DMEM, cat. # A14430-01), trypsin (cat. # 25300), penicillin-streptomycin solution (PenStrep, cat. # 15140-122), L-glutamine (L-Gln, cat. # 25030081), trypsin without phenol red (cat. # 15090046), and the BCA Protein Assay Kit (cat. # 23227) were purchased from Thermo Fisher Scientific (Waltham, MA, USA). The reagents 3-(4,5-dimethylthiazol-2-yl)-2,5-diphenyltetrazolium bromide (MTT; cat. # M2128), N, N-dimethylformamide (DMF; cat. # D4254), oligomycin (cat. # 75351), Carbonyl cyanide 4-(trifluoromethoxy) phenylhydrazone (FCCP; cat. # C2920), antimycin A (cat. # A8674), fetal bovine serum (FBS; cat. # F2442), and 2-deoxyglucose (2DG; cat. # D8375) were purchased from Sigma-Aldrich (St. Louis, MO, USA). Rotenone (cat. # 13995) was purchased from Cayman Chemical Company (Ann Arbor, MI, USA). Seahorse materials and reagents including 96-well cell culture plates and cartridges, calibration buffer, and XF Base media were purchased from Agilent (Santa Clara, CA, USA).

### Skin fibroblasts

Human skin fibroblasts derived from patients with MS were obtained from the Center of Multiple Sclerosis and Autoimmune Neurology at the Mayo Clinic (Rochester, MN, USA). Collection and storage of skin fibroblasts was performed at Mayo Clinic’s Center for Regenerative Medicine Biotrust. Skin fibroblasts from healthy individuals and patients with ALS were obtained from the Mayo Clinic’s Biotrust. The use of skin fibroblasts for these studies was approved by the Mayo Clinic Institutional Review Board. All cell lines used in this study were between passages six and ten. Skin fibroblasts were subcultured in MEM media supplemented with 10% FBS, 2 mM L-Gln, 1X NEAA, and 1X PenStrep maintained at 37 °C in a humidified chamber with 5% CO_2_/95% air. Supplementary Table 1 lists all human skin fibroblasts used in this study.

### Transmission electron microscopy

Specimens for electron microscopy imaging were prepared at the Microscopy and Cell Analysis Core at the Mayo Clinic. Briefly, skin fibroblasts were seeded onto a glass coverslip at 80,000 cells per well in a six-well plate in MEM media containing 10% FBS, L-Gln, NEAA, and PenStrep. Cells were grown for one week and refed on days three and six. Skin fibroblasts were fixed in 4% paraformaldehyde + 1% glutaraldehyde in 0.1 M phosphate buffer, pH 7.2 for 12 hours at 4 °C. Further processing was performed with the aid of a PELCO BioWave laboratory microwave oven (Ted Pella, Inc., Redding, CA) operating at 250W. Fixed cells were microwaved under vacuum in 1% osmium tetroxide in 0.1 M phosphate buffer for the sequence: 2 minutes on; 2 minutes off; 2 minutes on; 15 minutes off. Cells were rinsed in nH_2_O and microwaved in 2% uranyl acetate using the same sequence. Following another nH_2_O rinse, cells were dehydrated using the graded ethanol series (70%, 80%, 95%, 100%, 100% acetone). For each step in the dehydration, cells were microwaved for 40 seconds and allowed to rest at room temperature for 2 minutes. Cells were then infiltrated with Embed 812/Araldite resin (EMS, Hatfield, PA) by microwaving under vacuum 2 minutes on, 2 minutes off, 2 minutes on, 30 minutes off, in 2:1, 1:1 and 3:1 ratio of acetone:resin sequentially. After a final incubation in 100% resin for 12 hours at room temperature, cells were embedded by inverting resin filled embedding molds atop the monolayer allowing polymerization for 24 hours at 60 °C. Ultrathin sections (0.1 micron) were stained with lead citrate for 5 minutes at room temperature. Micrographs were acquired using a JEOL 1400+ Transmission Electron Microscope (JEOL, Inc., Peabody, MA) at 80 kV equipped with a Gatan Orius camera (Gatan, Inc., Warrendale, PA).

### Gene expression analysis

Skin fibroblasts were seeded at 80,000 cells per well in a six-well plate in MEM media containing 10% FBS, L-Gln, NEAA, and PenStrep. The cells were grown for one week and refed on day three and six. Media was removed and cells were rinsed with PBS. Total RNA was isolated using an RNeasy Mini Kit (Qiagen, Hilden, Germany, cat. # 74106). Purified RNA was eluted in water and the concentration was determined with a NanoDrop 2000 (Thermo Fisher Scientific). Total purified RNA (250-500 ng) was reverse transcribed using the Transcriptor First Strand cDNA Synthesis Kit (Roche, Basel, Switzerland, cat. # 04379012001). The PCR reactions contained a total of 5 ng cDNA and 10 pmol of primer in Sso Advanced Universal SYBR Green Supermix (Bio-Rad, Hercules, CA, USA, cat. # 1725271). Target genes were monitored using the primer pairs listed in Supplementary Table 2. Real-time PCR was run on a BIORAD CFX Connect Optics Module Real-Time System. Thermal cycling settings included one denaturing cycle at 95 °C for 30 seconds, 50 amplification rounds segmented for 95 °C for 15 seconds and 55 °C for 45 seconds, and one cycle at 65 °C for five seconds followed by 95 °C for one second. Amplification was monitored by fluorescence and Ct values were determined using the CFX Manager 3.1 software (Bio-Rad). Gene expression levels were normalized to β-actin (ΔCt = Ct(target) – Ct(β-actin)). Relative changes in gene expression were calculated using the 2^-ΔΔCt^ after averaging all control cell lines (ΔΔCt = ΔCt(sample) – ΔCt(control, average)) [74]. Each sample was measured in duplicate. All technical and biological replicates were averaged for analysis.

### Gamma irradiation

Skin fibroblasts were seeded at 1.6 x 10^5^ cells per well in a six-well dish in MEM containing 10% FBS, L-Gln, NEAA, and PenStrep. The next day, cells were exposed to 30 gray of gamma irradiation (Mark 1 Irradiator with cesium 137 γ source, JL Shepherd and Associates, San Fernando, CA, USA) and incubated at 37 °C for an additional 10 days. Total RNA isolation and gene expression was determined as described in the gene expression analysis section. The primers are listed in Supplementary Table 2. Gene expression levels were normalized to TATA box protein (TBP) (ΔCt = Ct(target) – Ct(TBP)). Each sample was measured in duplicate. All technical and biological repeats were averaged for analysis.

### Cell toxicity assays with hydrogen peroxide

Cell toxicity was evaluated using the MTT reduction assay or CellTox Green Cytotoxicity assay (Promega, Madison, WI, USA, Kit #G8741). For the MTT assay, skin fibroblasts were seeded in duplicate at 20,000 cells per well in a 96-well plate in MEM containing 10% FBS, L-Gln, NEAA, and PenStrep, and grown overnight at 37 °C. The media was replaced with 100 μL fresh media (phenol-free) with or without 200 μM hydrogen peroxide and incubated for an additional two hours at 37 °C. At the end of treatment, 10 μL of MTT reagent (5 mg/mL) was added to each well for two hours at 37 °C. Reduced MTT reagent (formazan) was solubilized by adding 100 μL of Lysis Solution (20% SDS (w/v) and 50% DMF (v/v) in water) to the wells and incubating at 37 °C for 30 minutes with agitation. The MTT solution was mixed once more by pipetting and 150 μL was transferred to a new clear 96-well plate. The absorbance was measured at 570 nm using a Varioskan Lux Type 3020 plate reader (Thermo Fisher Scientific). The percent toxicity (100 x treated/untreated) was calculated from the average of all technical and biological repeats.

For the CellTox Green Cytotoxicity assay (Promega cat. # G8741), cells were plated and treated with hydrogen peroxide as described for the MTT assay. In addition, CellTox Green Dye was added to the media at a 1:500 dilution. For the positive control, Lysis Reagent (provided by the Promega kit) was added to cells at 1:25. Lysing cells enables maximal green fluorescence and was used to normalize data. The plate was scanned every hour up to ten hours post-treatment. Cells were maintained at 37 °C during the course of the assay. The first scan one hour post treatment was used to normalize toxicity calculations. All cells were tested in duplicate. The data is averaged from all technical and biological repeats presented as a percent viability (100 x (Total_GFP_ - Treated_GFP_)/Total_GFP_).

### Seahorse assays

Skin fibroblasts were seeded at 20,000 cells per well in an Agilent XF 96-well format in MEM containing 10% FBS, 2 mM L-Gln, PenStrep, and NEAA and incubated overnight at 37 °C. Media was removed and cells were rinsed once with pre-warmed XF-base medium (pH 7.4). For the glycolytic stress test, 125 μL of XF-base medium supplemented with 2 mM L-Gln was added to the cells followed by a one hour incubation in a non-CO_2_ humidified chamber. The cells were then loaded into the Agilent Seahorse Analyzer and glycolytic function was analyzed. After baseline measurements, cells were treated in sequence with 5.5 mM glucose, 1.5 μM oligomycin, and 50 mM 2-DG. Each set of measurements were taken with the following setup: mix for three minutes, measure for three minutes, repeat cycle three times. For the mitochondrial stress test, 125 μL of XF-base medium supplemented with 2 mM L-Gln and 5.5 mM glucose was added to the cells. The cells were then incubated in a non-CO_2_ chamber for one hour. The cells were loaded onto the Agilent Seahorse Analyzer and mitochondrial function was analyzed. The baseline parameters were measured followed by the sequential treatments of 1.5 μM oligomycin, 1 μM FCCP, and a combination of 1 μM rotenone plus 1 μM antimycin A. Measurements were taken as described for the glycolytic stress test. All cell lines were plated in triplicates. Each assay was run in duplicate. According to Agilent’s guidelines, zeros were removed prior to analysis. All remaining replicates were averaged for each cell line. Calculations were determined from the average of all three measurements per injection, and normalized to total protein content.

### Targeted metabolomics

Skin fibroblasts from eight control and eight multiple sclerosis individuals were seeded at 40,000 cells per well in a 96-well plate containing 200 μL MEM supplemented with 10% FBS, 2 mM L-Gln, NEAA, and PenStrep, and grown for 24 hours at 37 °C. The conditioned supernatant was collected in a 1.5 mL Eppendorf tube and stored at -80 °C. Cells were then rinsed with PBS and the protein content was measured using the Pierce BCA Protein Assay Kit (Thermo Fisher Scientific, cat. # 23225). For metabolomics analysis, samples were delivered to the Biochemical Genetics Laboratory at the Mayo Clinic for targeted metabolomics analysis by gas chromatography/mass spectrometry (GC/MS). Media was thawed and spiked with internal standards (Supplementary Table 3) followed by pentafluorobenzyl oxime derivatization. The samples were acidified with HCl and extracted into ethyl acetate. The samples were evaporated using a Caliper Turbo Vap LV concentrator followed by silylation with N,O,-bis-(trimethylsilyl) trifluoroacetamide and 1% trimethylchlorosilane (BSTFA+TMCS). Treated samples were analyzed by capillary GC/MS (Agilent 7890B) using selected ion monitoring with positive ammonia chemical ionization and stable isotope dilution. Data acquisition and quantitation was performed using Agilent Masshunter B.07. The detected metabolite concentrations were normalized to protein content. All samples were measured in quadruplicates and averaged for analysis.

### Lactate assay

Skin fibroblasts were seeded at 40,000 cells per well in a 96-well clear plate cultured in 200 μL of MEM containing 10% FBS for 24 hours or in MEM lacking FBS for 24 and 48 hours at 37 °C. Lactate concentration in the media was determined using the Lactate Assay Kit (Eton Bioscience Inc., San Diego, CA, USA, cat. # 120001100A). Briefly, 50 μL of media was mixed with 50 μL Lactate Assay Solution and incubated in the dark in a non-CO_2_ incubator at 37 °C for 30 minutes. The reaction was quenched by addition of 50 μL of 0.5 M acetic acid. Samples were measured on a Spectra Max M3 plate reader using absorbance 490 nm. A standard curve containing L-lactate was generated for each assay. Cells were rinsed with PBS and incubated with 20 μL of RIPA lysis buffer for 10 minutes. Protein lysate concentrations were determined using the Pierce BCA Protein Assay Kit (Thermo Fisher Scientific, cat. # 23225). Lactate concentrations were normalized to total protein content and sample volume (200 μL). Each skin fibroblast was analyzed in triplicate wells. All technical and biological replicates were averaged for analysis.

### Statistical analysis

In all experiments, skin fibroblasts were matched by age and gender (Supplementary Table 4). Please note that ALS skin fibroblasts were approximately five years older in age due to the average disease onset/diagnosis occurring later in life compared to MS. The generation of graphs and statistical analyses was performed using GraphPad Prism software version 8. Gene expression data, the MTT assay, Seahorse data where indicated, and Lactate Assay data was analyzed using one-way ANOVA post hoc Tukey two-tailed t test to determine significance between multiple groups. Paired Seahorse data was analyzed using two-tailed t test. The CellTox Green Assay was analyzed using two-way ANOVA post hoc Tukey test. Metabolomics data was analyzed using a two-tailed t test for the comparison between two groups.

## Supplementary Material

Supplementary Figure 1

Supplementary Tables

## References

[1] Ransohoff RM, Hafler DA, Lucchinetti CF. Multiple sclerosis-a quiet revolution. Nat Rev Neurol. 2015; 11:134–42. 10.1038/nrneurol.2015.14 25686758 PMC4556342

[2] Baranzini SE, Oksenberg JR. The genetics of multiple sclerosis: from 0 to 200 in 50 years. Trends Genet. 2017; 33:960–70. 10.1016/j.tig.2017.09.004 28987266 PMC5701819

[3] Hipp MS, Kasturi P, Hartl FU. The proteostasis network and its decline in ageing. Nat Rev Mol Cell Biol. 2019; 20:421–35. 10.1038/s41580-019-0101-y 30733602

[4] Hou Y, Dan X, Babbar M, Wei Y, Hasselbalch SG, Croteau DL, Bohr VA. Ageing as a risk factor for neurodegenerative disease. Nat Rev Neurol. 2019; 15:565–81. 10.1038/s41582-019-0244-7 31501588

[5] Høgestøl EA, Kaufmann T, Nygaard GO, Beyer MK, Sowa P, Nordvik JE, Kolskår K, Richard G, Andreassen OA, Harbo HF, Westlye LT. Cross-sectional and longitudinal MRI brain scans reveal accelerated brain aging in multiple sclerosis. Front Neurol. 2019; 10:450. 10.3389/fneur.2019.00450 31114541 PMC6503038

[6] Albanese M, Zagaglia S, Landi D, Boffa L, Nicoletti CG, Marciani MG, Mandolesi G, Marfia GA, Buttari F, Mori F, Centonze D. Cerebrospinal fluid lactate is associated with multiple sclerosis disease progression. J Neuroinflammation. 2016; 13:36. 10.1186/s12974-016-0502-1 26863878 PMC4750170

[7] Mháille AN, McQuaid S, Windebank A, Cunnea P, McMahon J, Samali A, FitzGerald U. Increased expression of endoplasmic reticulum stress-related signaling pathway molecules in multiple sclerosis lesions. J Neuropathol Exp Neurol. 2008; 67:200–11. 10.1097/NEN.0b013e318165b239 18344911

[8] Wentling M, Lopez-Gomez C, Park HJ, Amatruda M, Ntranos A, Aramini J, Petracca M, Rusielewicz T, Chen E, Tolstikov V, Kiebish M, Fossati V, Inglese M, et al. A metabolic perspective on CSF-mediated neurodegeneration in multiple sclerosis. Brain. 2019; 142:2756–74. 10.1093/brain/awz201 31305892

[9] Way SW, Popko B. Harnessing the integrated stress response for the treatment of multiple sclerosis. Lancet Neurol. 2016; 15:434–43. 10.1016/S1474-4422(15)00381-6 26873788 PMC4792730

[10] Oost W, Talma N, Meilof JF, Laman JD. Targeting senescence to delay progression of multiple sclerosis. J Mol Med (Berl). 2018; 96:1153–66. 10.1007/s00109-018-1686-x 30229272 PMC6208951

[11] Reich DS, Lucchinetti CF, Calabresi PA. Multiple sclerosis. N Engl J Med. 2018; 378:169–80. 10.1056/NEJMra1401483 29320652 PMC6942519

[12] Mincu RI, Magda SL, Mihaila S, Florescu M, Mihalcea DJ, Velcea A, Chiru A, Tiu C, Popescu BO, Cinteza M, Vinereanu D. Impaired cardiac function in patients with multiple sclerosis by comparison with normal subjects. Sci Rep. 2018; 8:3300. 10.1038/s41598-018-21599-0 29459794 PMC5818507

[13] Wens I, Dalgas U, Vandenabeele F, Krekels M, Grevendonk L, Eijnde BO. Multiple sclerosis affects skeletal muscle characteristics. PLoS One. 2014; 9:e108158. 10.1371/journal.pone.0108158 25264868 PMC4180259

[14] Gebregiworgis T, Nielsen HH, Massilamany C, Gangaplara A, Reddy J, Illes Z, Powers R. A urinary metabolic signature for multiple sclerosis and neuromyelitis optica. J Proteome Res. 2016; 15:659–66. 10.1021/acs.jproteome.5b01111 26759122 PMC4772670

[15] D’Ambrosio A, Pontecorvo S, Colasanti T, Zamboni S, Francia A, Margutti P. Peripheral blood biomarkers in multiple sclerosis. Autoimmun Rev. 2015; 14:1097–110. 10.1016/j.autrev.2015.07.014 26226413

[16] Amorini AM, Nociti V, Petzold A, Gasperini C, Quartuccio E, Lazzarino G, Di Pietro V, Belli A, Signoretti S, Vagnozzi R, Lazzarino G, Tavazzi B. Serum lactate as a novel potential biomarker in multiple sclerosis. Biochim Biophys Acta. 2014; 1842:1137–43. 10.1016/j.bbadis.2014.04.005 24726946

[17] Konrad C, Kawamata H, Bredvik KG, Arreguin AJ, Cajamarca SA, Hupf JC, Ravits JM, Miller TM, Maragakis NJ, Hales CM, Glass JD, Gross S, Mitsumoto H, Manfredi G. Fibroblast bioenergetics to classify amyotrophic lateral sclerosis patients. Mol Neurodegener. 2017; 12:76. 10.1186/s13024-017-0217-5 29065921 PMC5655870

[18] Teves JM, Bhargava V, Kirwan KR, Corenblum MJ, Justiniano R, Wondrak GT, Anandhan A, Flores AJ, Schipper DA, Khalpey Z, Sligh JE, Curiel-Lewandrowski C, Sherman SJ, Madhavan L. Parkinson’s disease skin fibroblasts display signature alterations in growth, redox homeostasis, mitochondrial function, and autophagy. Front Neurosci. 2018; 11:737. 10.3389/fnins.2017.00737 29379409 PMC5770791

[19] Jędrak P, Mozolewski P, Węgrzyn G, Więckowski MR. Mitochondrial alterations accompanied by oxidative stress conditions in skin fibroblasts of Huntington’s disease patients. Metab Brain Dis. 2018; 33:2005–17. 10.1007/s11011-018-0308-1 30120672 PMC6244791

[20] Pérez MJ, Ponce DP, Osorio-Fuentealba C, Behrens MI, Quintanilla RA. Mitochondrial bioenergetics is altered in fibroblasts from patients with sporadic Alzheimer’s disease. Front Neurosci. 2017; 11:553. 10.3389/fnins.2017.00553 29056898 PMC5635042

[21] Khan TK, Sen A, Hongpaisan J, Lim CS, Nelson TJ, Alkon DL. PKCε deficits in Alzheimer’s disease brains and skin fibroblasts. J Alzheimers Dis. 2015; 43:491–509. 10.3233/JAD-141221 25125477

[22] Joachim CL, Mori H, Selkoe DJ. Amyloid beta-protein deposition in tissues other than brain in Alzheimer’s disease. Nature. 1989; 341:226–30. 10.1038/341226a0 2528696

[23] Johnston JA, Cowburn RF, Norgren S, Wiehager B, Venizelos N, Winblad B, Vigo-Pelfrey C, Schenk D, Lannfelt L, O’Neill C. Increased beta-amyloid release and levels of amyloid precursor protein (APP) in fibroblast cell lines from family members with the Swedish Alzheimer’s disease APP670/671 mutation. FEBS Lett. 1994; 354:274–78. 10.1016/0014-5793(94)01137-0 7957938

[24] Szelechowski M, Amoedo N, Obre E, Léger C, Allard L, Bonneu M, Claverol S, Lacombe D, Oliet S, Chevallier S, Le Masson G, Rossignol R. Metabolic reprogramming in amyotrophic lateral sclerosis. Sci Rep. 2018; 8:3953. 10.1038/s41598-018-22318-5 29500423 PMC5834494

[25] Kirk K, Gennings C, Hupf JC, Tadesse S, D’Aurelio M, Kawamata H, Valsecchi F, Mitsumoto H, Manfredi G, and ALS/PLS COSMOS Study Groups. Bioenergetic markers in skin fibroblasts of sporadic amyotrophic lateral sclerosis and progressive lateral sclerosis patients. Ann Neurol. 2014; 76:620–24. 10.1002/ana.24244 25090982 PMC4192005

[26] Bernales S, McDonald KL, Walter P. Autophagy counterbalances endoplasmic reticulum expansion during the unfolded protein response. PLoS Biol. 2006; 4:e423. 10.1371/journal.pbio.0040423 17132049 PMC1661684

[27] van Deursen JM. The role of senescent cells in ageing. Nature. 2014; 509:439–46. 10.1038/nature13193 24848057 PMC4214092

[28] Haronikova L, Olivares-Illana V, Wang L, Karakostis K, Chen S, Fåhraeus R. The p53 mRNA: an integral part of the cellular stress response. Nucleic Acids Res. 2019; 47:3257–71. 10.1093/nar/gkz124 30828720 PMC6468297

[29] Baker DJ, Petersen RC. Cellular senescence in brain aging and neurodegenerative diseases: evidence and perspectives. J Clin Invest. 2018; 128:1208–16. 10.1172/JCI95145 29457783 PMC5873891

[30] Tacutu R, Budovsky A, Yanai H, Fraifeld VE. Molecular links between cellular senescence, longevity and age-related diseases - a systems biology perspective. Aging (Albany NY). 2011; 3:1178–91. 10.18632/aging.100413 22184282 PMC3273898

[31] Childs BG, Durik M, Baker DJ, van Deursen JM. Cellular senescence in aging and age-related disease: from mechanisms to therapy. Nat Med. 2015; 21:1424–35. 10.1038/nm.4000 26646499 PMC4748967

[32] Nicaise AM, Wagstaff LJ, Willis CM, Paisie C, Chandok H, Robson P, Fossati V, Williams A, Crocker SJ. Cellular senescence in progenitor cells contributes to diminished remyelination potential in progressive multiple sclerosis. Proc Natl Acad Sci USA. 2019; 116:9030–39. 10.1073/pnas.1818348116 30910981 PMC6500153

[33] Kritsilis M, V Rizou S, Koutsoudaki PN, Evangelou K, Gorgoulis VG, Papadopoulos D. Ageing, cellular senescence and neurodegenerative disease. Int J Mol Sci. 2018; 19:2937. 10.3390/ijms19102937 30261683 PMC6213570

[34] Li M, You L, Xue J, Lu Y. Ionizing radiation-induced cellular senescence in normal, non-transformed cells and the involved DNA damage response: a mini review. Front Pharmacol. 2018; 9:522. 10.3389/fphar.2018.00522 29872395 PMC5972185

[35] Sharpless NE, Sherr CJ. Forging a signature of in vivo senescence. Nat Rev Cancer. 2015; 15:397–408. 10.1038/nrc3960 26105537

[36] Mahad DH, Trapp BD, Lassmann H. Pathological mechanisms in progressive multiple sclerosis. Lancet Neurol. 2015; 14:183–93. 10.1016/S1474-4422(14)70256-X 25772897

[37] Lassmann H, van Horssen J. Oxidative stress and its impact on neurons and glia in multiple sclerosis lesions. Biochim Biophys Acta. 2016; 1862:506–10. 10.1016/j.bbadis.2015.09.018 26432481

[38] Liu Y, Adachi M, Zhao S, Hareyama M, Koong AC, Luo D, Rando TA, Imai K, Shinomura Y. Preventing oxidative stress: a new role for XBP1. Cell Death Differ. 2009; 16:847–57. 10.1038/cdd.2009.14 19247368 PMC2826168

[39] Berridge MV, Tan AS. Characterization of the cellular reduction of 3-(4,5-dimethylthiazol-2-yl)-2,5-diphenyltetrazolium bromide (MTT): subcellular localization, substrate dependence, and involvement of mitochondrial electron transport in MTT reduction. Arch Biochem Biophys. 1993; 303:474–82. 10.1006/abbi.1993.1311 8390225

[40] Riss T, Niles A, Moravec R, Karassina N, Vidugiriene J. Cytotoxicity Assays: In Vitro Methods to Measure Dead Cells. 2019 May 1. In: Sittampalam GS, Grossman A, Brimacombe K, Arkin M, Auld D, Austin CP, Baell J, Bejcek B, Caaveiro JMM, Chung TDY, Coussens NP, Dahlin JL, Devanaryan V, Foley TL, Glicksman M, Hall MD, et al., editors. Assay Guidance Manual [Internet]. Bethesda (MD): Eli Lilly & Company and the National Center for Advancing Translational Sciences; 2004. 31070879

[41] Metallo CM, Vander Heiden MG. Understanding metabolic regulation and its influence on cell physiology. Mol Cell. 2013; 49:388–98. 10.1016/j.molcel.2013.01.018 23395269 PMC3569837

[42] Barcelos IP, Troxell RM, Graves JS. Mitochondrial dysfunction and multiple sclerosis. Biology (Basel). 2019; 8:37. 10.3390/biology8020037 31083577 PMC6627385

[43] Adiele RC, Adiele CA. Metabolic defects in multiple sclerosis. Mitochondrion. 2019; 44:7–14. 10.1016/j.mito.2017.12.005 29246870

[44] Divakaruni AS, Paradyse A, Ferrick DA, Murphy AN, Jastroch M. Analysis and interpretation of microplate-based oxygen consumption and pH data. Methods Enzymol. 2014; 547:309–54. 10.1016/B978-0-12-801415-8.00016-3 25416364

[45] Blackburn PR, Gass JM, Vairo FP, Farnham KM, Atwal HK, Macklin S, Klee EW, Atwal PS. Maple syrup urine disease: mechanisms and management. Appl Clin Genet. 2017; 10:57–66. 10.2147/TACG.S125962 28919799 PMC5593394

[46] Disanto G, Berlanga AJ, Handel AE, Para AE, Burrell AM, Fries A, Handunnetthi L, De Luca GC, Morahan JM. Heterogeneity in multiple sclerosis: scratching the surface of a complex disease. Autoimmune Dis. 2010; 2011:932351. 10.4061/2011/932351 21197462 PMC3005811

[47] Schröder M, Kaufman RJ. The mammalian unfolded protein response. Annu Rev Biochem. 2005; 74:739–89. 10.1146/annurev.biochem.73.011303.074134 15952902

[48] Zhu G, Lee AS. Role of the unfolded protein response, GRP78 and GRP94 in organ homeostasis. J Cell Physiol. 2015; 230:1413–20. 10.1002/jcp.24923 25546813 PMC4725317

[49] Hiramatsu N, Messah C, Han J, LaVail MM, Kaufman RJ, Lin JH. Translational and posttranslational regulation of XIAP by eIF2α and ATF4 promotes ER stress-induced cell death during the unfolded protein response. Mol Biol Cell. 2014; 25:1411–20. 10.1091/mbc.E13-11-0664 24623724 PMC4004591

[50] Grootjans J, Kaser A, Kaufman RJ, Blumberg RS. The unfolded protein response in immunity and inflammation. Nat Rev Immunol. 2016; 16:469–84. 10.1038/nri.2016.62 27346803 PMC5310224

[51] Kraus A, Michalak M. Endoplasmic reticulum quality control and dysmyelination. Biomol Concepts. 2011; 2:261–74. 10.1515/bmc.2011.028 25962034

[52] Lin W, Bailey SL, Ho H, Harding HP, Ron D, Miller SD, Popko B. The integrated stress response prevents demyelination by protecting oligodendrocytes against immune-mediated damage. J Clin Invest. 2007; 117:448–56. 10.1172/JCI29571 17273557 PMC1783809

[53] Roussel BD, Kruppa AJ, Miranda E, Crowther DC, Lomas DA, Marciniak SJ. Endoplasmic reticulum dysfunction in neurological disease. Lancet Neurol. 2013; 12:105–18. 10.1016/S1474-4422(12)70238-7 23237905

[54] Karamita M, Nicholas R, Kokoti L, Rizou S, Mitsikostas DD, Gorgoulis V, Probert L, Papadopoulos D. Cellular Senescence Correlates with Demyelination, Brain Atrophy and Motor Impairment in a Model of Multiple Sclerosis (P2.405). Neurology. 2018; 90:P2.405.

[55] Ohl K, Tenbrock K, Kipp M. Oxidative stress in multiple sclerosis: central and peripheral mode of action. Exp Neurol. 2016; 277:58–67. 10.1016/j.expneurol.2015.11.010 26626971 PMC7094520

[56] Sprenkle NT, Sims SG, Sánchez CL, Meares GP. Endoplasmic reticulum stress and inflammation in the central nervous system. Mol Neurodegener. 2017; 12:42. 10.1186/s13024-017-0183-y 28545479 PMC5445486

[57] Wiley CD, Campisi J. From ancient pathways to aging cells-connecting metabolism and cellular senescence. Cell Metab. 2016; 23:1013–21. 10.1016/j.cmet.2016.05.010 27304503 PMC4911819

[58] Peruzzotti-Jametti L, Pluchino S. Targeting mitochondrial metabolism in neuroinflammation: towards a therapy for progressive multiple sclerosis. Trends Mol Med. 2018; 24:838–55. 10.1016/j.molmed.2018.07.007 30100517

[59] Gaber T, Strehl C, Buttgereit F. Metabolic regulation of inflammation. Nat Rev Rheumatol. 2017; 13:267–79. 10.1038/nrrheum.2017.37 28331208

[60] Tannahill GM, Iraci N, Gaude E, Frezza C, Pluchino S. Metabolic reprograming of mononuclear phagocytes in progressive multiple sclerosis. Front Immunol. 2015; 6:106. 10.3389/fimmu.2015.00106 25814990 PMC4356156

[61] Klotz L, Eschborn M, Lindner M, Liebmann M, Herold M, Janoschka C, Torres Garrido B, Schulte-Mecklenbeck A, Gross CC, Breuer J, Hundehege P, Posevitz V, Pignolet B, et al. Teriflunomide treatment for multiple sclerosis modulates T cell mitochondrial respiration with affinity-dependent effects. Sci Transl Med. 2019; 11:eaao5563. 10.1126/scitranslmed.aao5563 31043571

[62] Mooney BP, Miernyk JA, Randall DD. The complex fate of alpha-ketoacids. Annu Rev Plant Biol. 2002; 53:357–75. 10.1146/annurev.arplant.53.100301.135251 12221980

[63] Bridi R, Braun CA, Zorzi GK, Wannmacher CM, Wajner M, Lissi EG, Dutra-Filho CS. Alpha-keto acids accumulating in maple syrup urine disease stimulate lipid peroxidation and reduce antioxidant defences in cerebral cortex from young rats. Metab Brain Dis. 2005; 20:155–67. 10.1007/s11011-005-4152-8 15938133

[64] Funchal C, Latini A, Jacques-Silva MC, Dos Santos AQ, Buzin L, Gottfried C, Wajner M, Pessoa-Pureur R. Morphological alterations and induction of oxidative stress in glial cells caused by the branched-chain alpha-keto acids accumulating in maple syrup urine disease. Neurochem Int. 2006; 49:640–50. 10.1016/j.neuint.2006.05.007 16822590

[65] Jan W, Zimmerman RA, Wang ZJ, Berry GT, Kaplan PB, Kaye EM. MR diffusion imaging and MR spectroscopy of maple syrup urine disease during acute metabolic decompensation. Neuroradiology. 2003; 45:393–99. 10.1007/s00234-003-0955-7 12736767

[66] Yudkoff M, Daikhin Y, Nissim I, Horyn O, Luhovyy B, Luhovyy B, Lazarow A, Nissim I. Brain amino acid requirements and toxicity: the example of leucine. J Nutr. 2005; 135:1531S–8S. 10.1093/jn/135.6.1531S 15930465

[67] Kane DA. Lactate oxidation at the mitochondria: a lactate-malate-aspartate shuttle at work. Front Neurosci. 2014; 8:366. 10.3389/fnins.2014.00366 25505376 PMC4243568

[68] Harjes U. Metabolism: more lactate, please. Nat Rev Cancer. 2017; 17:707. 10.1038/nrc.2017.101 29077692

[69] Tauffenberger A, Fiumelli H, Almustafa S, Magistretti PJ. Lactate and pyruvate promote oxidative stress resistance through hormetic ROS signaling. Cell Death Dis. 2019; 10:653. 10.1038/s41419-019-1877-6 31506428 PMC6737085

[70] Zelenka J, Dvořák A, Alán L. L-lactate protects skin fibroblasts against aging-associated mitochondrial dysfunction via mitohormesis. Oxid Med Cell Longev. 2015; 2015:351698. 10.1155/2015/351698 26171114 PMC4478408

[71] Latham T, Mackay L, Sproul D, Karim M, Culley J, Harrison DJ, Hayward L, Langridge-Smith P, Gilbert N, Ramsahoye BH. Lactate, a product of glycolytic metabolism, inhibits histone deacetylase activity and promotes changes in gene expression. Nucleic Acids Res. 2012; 40:4794–803. 10.1093/nar/gks066 22323521 PMC3367171

[72] Hoque R, Farooq A, Ghani A, Gorelick F, Mehal WZ. Lactate reduces liver and pancreatic injury in toll-like receptor- and inflammasome-mediated inflammation via GPR81-mediated suppression of innate immunity. Gastroenterology. 2014; 146:1763–74. 10.1053/j.gastro.2014.03.014 24657625 PMC4104305

[73] Yang S, Zhang KY, Kariawasam R, Bax M, Fifita JA, Ooi L, Yerbury JJ, Nicholson GA, Blair IP. Evaluation of skin fibroblasts from amyotrophic lateral sclerosis patients for the rapid study of pathological features. Neurotox Res. 2015; 28:138–46. 10.1007/s12640-015-9532-1 26013250

[74] Livak KJ, Schmittgen TD. Analysis of relative gene expression data using real-time quantitative PCR and the 2(-delta delta C(T)) method. Methods. 2001; 25:402–08. 10.1006/meth.2001.1262 11846609

