# Correction for: Signatures of cell stress and altered bioenergetics in skin fibroblasts from patients with multiple sclerosis

**DOI:** 10.18632/aging.202757

**Published:** 2021-03-03

**Authors:** Jordan M. Wilkins, Oleksandr Gakh, Parijat Kabiraj, Christina B. McCarthy, W. Oliver Tobin, Charles L. Howe, Claudia F. Lucchinetti

**Affiliations:** 1 Department of Neurology, Mayo Clinic, Rochester, MN 55905, USA; 2 Department of Immunology, Mayo Clinic, Rochester, MN 55905, USA; 3 Center for Multiple Sclerosis and Autoimmune Neurology, Mayo Clinic, Rochester, MN 55905, USA

**Keywords:** correction

Original article: Aging. 2020; 12:15134–15156.  . https://doi.org/10.18632/aging.103612

**This article has been corrected:** The authors requested to add one more author to the authors list and remove name of this author from the **Acknowledgments**.

The correct authors list is given below:

Jordan M. Wilkins^1,*^, Oleksandr Gakh^1,*^, Parijat Kabiraj^1^, Christina B. McCarthy^1^, W. Oliver Tobin^1^, Charles L. Howe^1,2,3^, Nathan P. Staff^1^, Claudia F. Lucchinetti^1^

The correct **Acknowledgments** text is given below:

We thank Jessica Sagen and the CMSAN Biorepository for support and help acquiring MS skin fibroblasts, Stephen Weigand for statistical help, and Devin Oglesbee’s group for performing the metabolomics.

