# Supplementary material for: Signatures of cell stress and altered bioenergetics in skin fibroblasts from patients with multiple sclerosis
Source: Aging (Albany NY). 2020 Jul 8;12(14):15134–56. doi: 10.18632/aging.103612 (PMC7425440; doi:10.18632/aging.103612)
Supplement: Supplementary Figure 1 [file aging-12-103612-s001..pdf]

SUPPLEMENTARY FIGURE

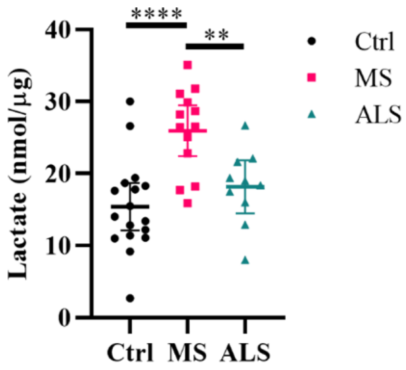

Supplementary Figure 1. Lactate production is increased in MS skin fibroblasts. Skin fibroblasts from MS (n = 13), ALS (n = 10), and control (n = 17) cells were incubated in media containing FBS for 24 hours. The lactate concentration in the spent media was determined and normalized to protein concentration (nmol lactate per µg protein). Significant changes between groups were determined using one-way ANOVA post hoc Tukey test. Each data point represents a unique skin fibroblast sample. The average of replicates is shown with the 95% confidence interval. **, P < 0.01; ****, P < 0.0001. Abbreviations: ALS, amyotrophic lateral sclerosis; Ctrl, control; MS, multiple sclerosis.
