# Supplementary material for: Signatures of cell stress and altered bioenergetics in skin fibroblasts from patients with multiple sclerosis
Source: Aging (Albany NY). 2020 Jul 8;12(14):15134–56. doi: 10.18632/aging.103612 (PMC7425440; doi:10.18632/aging.103612)
Supplement: Supplementary Tables [file aging-12-103612-s002..pdf]

SUPPLEMENTARY TABLES

Supplementary Table 1. Skin fibroblasts used in study.

Biotrust ID	Age (yr)	Sex	Diagnosis[a]	Duration (yr)[b]
011-BIOTR-0024	53	Male	Control	NA
014-BIOTR-0015	38	Male	Control	NA
014-BIOTR-0018	38	Female	Control	NA
014-BIOTR-0022	33	Female	Control	NA
014-BIOTR-0025	51	Female	Control	NA
014-BIOTR-0026	52	Female	Control	NA
014-BIOTR-0028	39	Female	Control	NA
014-BIOTR-0033	25	Male	Control	NA
100-BIOTR-0012	54	Female	Control	NA
100-BIOTR-0020	44	Male	Control	NA
100-BIOTR-0022	30	Male	Control	NA
100-BIOTR-0023	54	Female	Control	NA
100-BIOTR-0024	25	Male	Control	NA
100-BIOTR-0026	65	Female	Control	NA
100-BIOTR-0027	59	Male	Control	NA
100-BIOTR-0033	68	Male	Control	NA
100-BIOTR-0034	70	Female	Control	NA
100-BIOTR-0038	28	Male	Control	NA
100-BIOTR-0039	66	Male	Control	NA
100-BIOTR-0040	74	Female	Control	NA
100-BIOTR-0042	48	Male	Control	NA
100-BIOTR-0044	33	Male	Control	NA
100-BIOTR-0045	34	Male	Control	NA
100-BIOTR-0046	37	Female	Control	NA
019-BIOTR-0001	31	Male	RRMS	2.6
019-BIOTR-0002	46	Male	RRMS	2.2
019-BIOTR-0009	32	Female	RRMS	3.1
019-BIOTR-0014	39	Female	RRMS	3.4
019-BIOTR-0019	51	Female	RRMS	10.1
019-BIOTR-0021	66	Female	RRMS	1.9
019-BIOTR-0026	50	Female	RRMS	29.3
019-BIOTR-0028	44	Male	RRMS	0.2
019-BIOTR-0029	59	Male	RRMS	16.7
019-BIOTR-0030	70	Female	SPMS	14.9
019-BIOTR-0031	66	Female	RRMS	7.8
019-BIOTR-0034	62	Female	RRMS	20.5
019-BIOTR-0035	69	Female	RRMS	20.4
019-BIOTR-0036	54	Female	RRMS	12.5

019-BIOTR-0037	18	Female	RRMS	0.7
019-BIOTR-0040	36	Male	RRMS	1.5
019-BIOTR-0041	39	Male	RRMS	1.9
019-BIOTR-0045	43	Female	RRMS	0.4
019-BIOTR-0050	43	Female	CIS	0.8
019-BIOTR-0054	49	Male	RRMS	4.0
019-BIOTR-0055	72	Male	RRMS	11.5
019-BIOTR-0061	56	Male	RRMS	10.7
019-BIOTR-0063	46	Female	RRMS	20.8
019-BIOTR-0068	50	Male	RRMS	14.3
019-BIOTR-0069	30	Female	RRMS	0.3
019-BIOTR-0072	40	Female	RRMS	0.7
019-BIOTR-0073	39	Male	RRMS	2.4
019-BIOTR-0075	38	Female	RRMS	1.5
019-BIOTR-0082	54	Female	RRMS	0.1
019-BIOTR-0083	56	Female	SPMS	8.6
009-BIOTR-0013	62	Female	ALS	1.6
009-BIOTR-0017	56	Female	ALS	6.1
009-BIOTR-0018	42	Female	ALS	0.9
009-BIOTR-0020	33	Female	ALS	2.7
009-BIOTR-0029	52	Male	ALS	2.0
009-BIOTR-0032	63	Male	ALS	0.9
009-BIOTR-0033	45	Male	ALS	1.8
009-BIOTR-0038	71	Male	ALS	3.0
009-BIOTR-0059	72	Female	ALS	4.0
009-BIOTR-0060	36	Male	ALS	2.5

[a]Diagnosis at time of skin fibroblast harvest.
[b]Time in years from disease onset to skin fibroblast harvest.
Abbreviations: ALS, amyotrophic lateral sclerosis; CIS, clinically isolated syndrome; NA, not applicable; RRMS, relapsing-remitting multiple sclerosis; SPMS, secondary progressive multiple sclerosis; yr, years.

Supplementary Table 2. Sequence of RT-PCR primers.

Gene	Forward Primer	Reverse Primer
ACTB	GCCAACACAGTGCTGTCTGG	CTGCTTGCTGATCCACATCTGC
ATF4	GTTCTCCAGCGACAAGGCTA	ATCCTGCTTGCTGTTGTTGG
BIP	TGTTCAACCAATTATCAGCAAACTC	TTCTGCTGTATCCTCTTCACCAGT
CHOP	AGAACCAGGAAACGGAAACAGA	TCTCCTTCATGCGCTGCTTT
GRP94	GAAACGGATGCCTGGTGG	GCCCCTTCTTCCTGGGTC
P16	GTGAGAGTGGCGGGGTC	CCCAACGCACCGAATAGTTA
P21	GCCATTAGCGCATCACAGT	ACCGAGGCACTCAGAGGAG
P53	CTGCCCTCAACAAGATGTTTTG	CGGGCAGCTTCCTCTATGTTTATGTTATC
TBP	GCCAGCTTCGGAGAGTTCTGGGATT	CGGGCACGAAGTGCAATGGTCTTTA

Abbreviations: *ACTB*, actin beta; *ATF4*, activating transcription factor 4; *BIP*, heat shock protein family A member 5, HSPA5/BIP; *CHOP*, DNA damage inducible transcript 3, DDIT3/CHOP; *GRP94*, heat shock protein 90 beta family member 1, HSP90B1/GRP94; *P16*, cyclin dependent kinase inhibitor 2A, CDKN2A/P16; *P21*, cyclin dependent kinase inhibitor 1A, CDKN1A/P21; *P53*, tumor protein p53; *TBP*, TATA box protein.

Supplementary Table 3. Metabolites and standards analyzed by mass spectrometry.

Common name	Company	Cat. #	HMDB
L-Lactic acid	Sigma	71720	HMDB0000190
Pyruvic acid	Sigma	107360	HMDB0000243
2-Hydroxybutyric acid	Sigma	54917	HMDB0000008
3-Hydroxybutyric acid	Fisher Scientific	AC121050100	HMDB0000357
2-Ketobutyric acid	Sigma	K401	HMDB0000005
alpha-Ketoisovaleric acid	Sigma	198994	HMDB0000019
3-Methyl-2-oxovaleric acid	Sigma	K7125	HMDB0000491
Ketoleucine	Sigma	68255	HMDB0000695
3-Hydroxyisovaleric acid	VU Medical Center (Ten Brink)	4914	HMDB0000754
Acetoacetic acid	Sigma	A8509	HMDB0000060
Succinic acid	Sigma	S3674	HMDB0000254
Fumaric acid	Sigma	47910	HMDB0000134
3-Methylglutaconic acid	Sigma	06689	HMDB0000522
Malic acid	Sigma	240176	HMDB0000744
Oxoglutaric acid	Sigma	K1750	HMDB0000208
Citric acid	Sigma	C0759	HMDB0000094
cis-Aconitic acid	Sigma	A3412	HMDB0000072
	CDN Isotopes	D-2273	
	Sigma	490717	
	Sigma	488895	
	Cambridge Isotope Laboratories	DLM-3487	
	Cambridge Isotope Laboratories	CLM-6820	
	Cambridge Isotope Laboratories	CLM-4418	
	Cambridge Isotope Laboratories	CLM-1084	
	Cambridge Isotope Laboratories	CLM-1529	
	Sigma	702250	
	CDN Isotopes	D-2122	
	Cambridge Isotope Laboratories	CLM-4442	
	VU Medical Center (Ten Brink)	4910	

Abbreviations: HMDB, human metabolome database.

Supplementary Table 4. Summary of skin fibroblasts used in each assay.

Assay	Status	Number	% Female	Average age (years)	SD	Two-sample t-test[a]	Fishers exact test[b]
Markers *BIP*, *CHOP*, *ATF4*, *GRP94*, and *P53*	Control	20	50.0	46.8	14.6	0.66	1.0
	MS	22	54.5	44.9	12.9		
	ALS	9	44.4	52.9	14.8		
Markers *P16* and *P21*	Control	13	53.8	48.3	16.9	0.93	0.51
	MS	26	65.4	47.8	13.9		
	ALS	10	50.0	53.2	13.9		
MTT assay	Ctrl	16	56.3	49.2	13.1	0.61	1.0
	MS	10	50.0	47.1	7.8		
	ALS	10	50.0	53.2	13.9		
CellTox assay	Ctrl	13	53.8	49.4	11.4	0.92	1.0
	MS	8	50.0	49.0	7.6		
	ALS	10	50.0	53.2	13.9		
Seahorse assay	Ctrl	8	50.0	51.0	15.5	0.87	1.0
	MS	8	50.0	52.3	15.5		
	ALS	8	50.0	53.0	15.8		
Lactate assay, 24 hours without FBS	Control	21	47.6	48.0	14.6	0.84	0.39
	MS	29	62.1	48.8	13.1		
	ALS	10	50.0	53.2	13.9		
Lactate assay, 48 hours without FBS	Control	20	50.0	49.1	14.0	0.45	0.77
	MS	25	56.0	46.0	12.6		
	ALS	9	44.4	52.9	14.8		
Lactate assay, 24 hours with FBS	Control	17	52.9	49.8	15.5	0.65	1.0
	MS	13	61.5	47.5	12.2		
	ALS	10	50.0	53.2	13.9		

[a]Significance for group comparisons (MS vs control) was calculated using two-sample t-test (two-tailed).
[b]Fisher's exact test was used to determine significance for categorical variables (MS vs control).
Abbreviations: ALS, amyotrophic lateral sclerosis; ATF4, activating transcription factor 4; BIP, heat shock protein family A member 5, HSPA5/BIP; CHOP, DNA damage inducible transcript 3, DDIT3/CHOP; FBS, fetal bovine serum; GRP94, heat shock protein 90 beta family member 1, HSP90B1/GRP94; MS, multiple sclerosis; MTT, 3-(4,5-dimethylthiazol-2-yl)-2,5-diphenyltetrazolium bromide; P16, cyclin dependent kinase inhibitor 2A, CDKN2A/P16; P21, cyclin dependent kinase inhibitor 1A, CDKN1A/P21; P53, tumor protein p53; and SD, standard deviation.
